# Supplementary material for: Ultrafine-Grained Zn–Mg–Sr Alloy Synthesized by Mechanical Alloying and Spark Plasma Sintering
Source: Materials (Basel). 2022 Nov 24;15(23):8379. doi: 10.3390/ma15238379 (PMC9736596; doi:10.3390/ma15238379)
Supplement: Supplementary file 1 [file materials-15-08379-s001.zip › materials-1991175-supplementary.pdf]

*Supplementary Material*

# Ultrafine-Grained Zn–Mg–Sr Alloy Synthesized by Mechanical Alloying and Spark Plasma Sintering

**David Nečas [1,*], Jiří Kubásek [1,*], Jan Pinc [2], Ivo Marek [1], Črtomir Donik [3], Irena Paulin [3] and Dalibor Vojtěch [1]**

[1] Department of Metals and Corrosion Engineering, Faculty of Chemical Technology, University of Chemistry and Technology, Prague Technická 5, 166 28, Praha 6–Dejvice, Czech Republic

[2] Department of Functional Materials, Institute of Physics of the Czech Academy of Sciences, Na Slovance 1999/2, 182 21 Prague 8, Czech Republic

[3] Department Physics and Chemistry of Materials, Institute of Metals and Technology, University of Ljubljana, Lepi pot 11, SI-1000 Ljubljana, Slovenia

* Correspondence: necasd@vscht.cz (D.N.); kubasekj@vscht.cz (J.K.)

## 1. Microstructure

Materials prepared by powder metallurgy are generally characterized by significantly different microstructures compared to conventionally prepared alloys. To clarify this statement, we show the microstructure of Zn-1Mg-0.5Sr alloy prepared by casting in Figure S1. The alloy was prepared by melting in a resistance furnace at 550 °C from pure Zn, Mg and Sr and cast into the non-preheated steel mould 50 mm in diameter. The exact composition corresponded to the Zn-0.9Mg-0.4Sr (according to Atomic absorption spectroscopy - AAS), which is similar to the materials prepared by powder metallurgy.

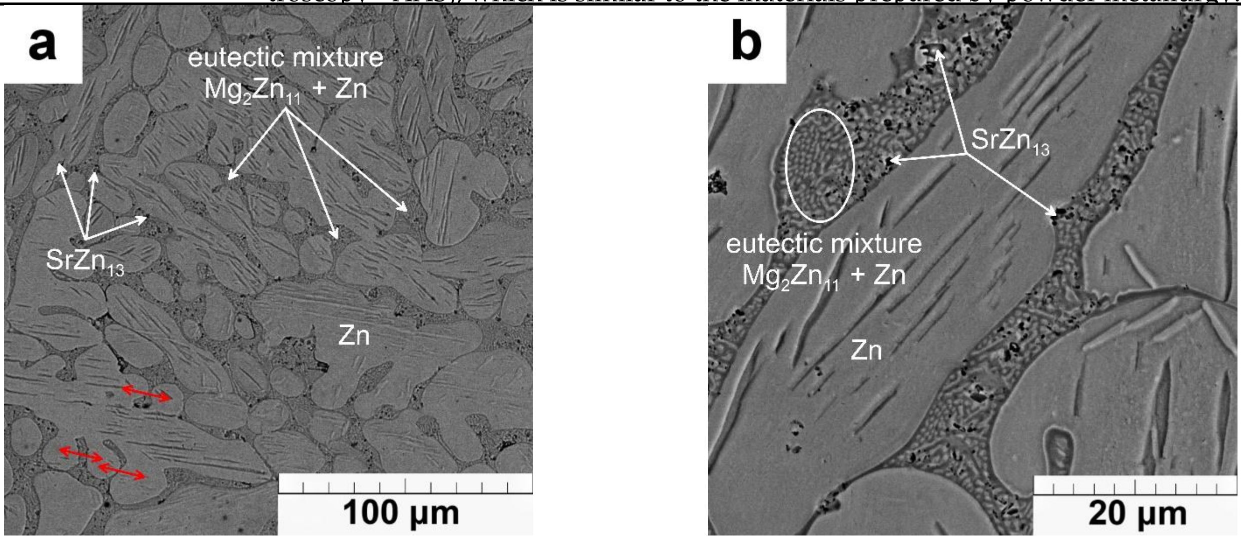


**Figure S1** Microstructure of Zn-0.9Mg-0.4Sr alloy prepared by casting - SEM: (a) overview, (b) detail.

The as-casted alloy is characterized by coarse microstructure with the average secondary dendrite arm spacing (SDAS) equal to 23 µm. This parameter was evaluated instead of grain size due to the character of the microstructure. The example of measurement is shown in Figure S1a in red lines. Primary dendrites are composed of pure Zn and minority amount of dissolved Mg (up to 0.1 wt.% according to Energy dispersive X-ray spectroscopy - EDS), while the Zn+$Mg_2Zn_{11}$ eutectic mixture occupies large areas at their interface. This mixture further contains the $SrZn_{13}$ intermetallic phases, which are very prone to dissolution, and therefore, are partially dissolved and removed during sample preparation for microstructure observation. Therefore, the black areas are the result of phase etching. The size of $SrZn_{13}$ differed in the range between 300 nm and 3 µm.

## 2. Mechanical properties

The mechanical properties of as-casted Zn-1Mg-0.5Sr were analysed like in the case of powder metallurgy products by hardness measurements (at least 10 measurements)

and compression tests (3 samples). The results are shown in Figure S2 and Table S1. As-casted material was characterized by a 32 % lower compressive yield strength (CYS) value (221 MPa) compared to the product of powder metallurgy. The hardness value is rather similar for both Zn-1Mg-0.5Sr materials, which is related to the presence of a similar amount of hard intermetallic phases like $Mg_2Zn_{11}$, $SrZn_{13}$, although in one case (as-cast material) as a eutectic mixture and in the second (powder metallurgy - PM) like distribution of fine intermetallics in zin matrix. Although the reader can see some differences between materials in the elastic part of deformation (Figure S2), measurements were not performed using the extensometer, so the differences in the elastic behaviour can not be considered properly. The as-casted alloy was deformed without the fracture, disabling the possibility of evaluation of ultimate compressive strength. This was similar to the case of powder metallurgy products.

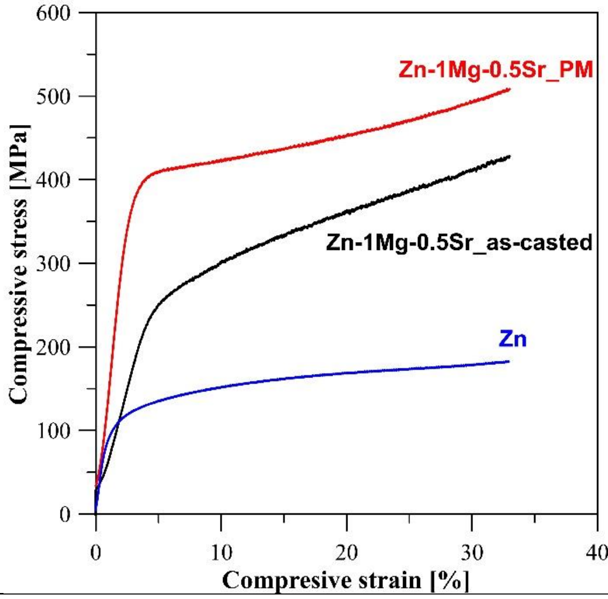


**Figure S2** Compressive stress-strain curves of materials prepared by powder metallurgy and as-casted Zn-1Mg-0.5Sr alloy.

**Table S1.** Mechanical properties of studied materials.

| Composition | Synthesis | Grain size [μm] | Hardness (HV1) | CYS [MPa] |
|---|---|---|---|---|
| Zn | MA+PM | - | 38±2 | 118±2 |
| Zn-1Mg-0.5Sr | MA+PM | 0.5 | 86±2 | 327±3 |
| Zn-1Mg-0.5Sr | As-casted | 126* | 82±4 | 221±6 |

*Secondary dendrite arm spacing was evaluated as the parameter of the microstructure coarseness.
